# Deleterious effects of severe sepsis and septic shock on physical activity in daily life, muscle strength and exercise capacity: a prospective cohort study

**DOI:** 10.1186/cc14712

**Published:** 2015-09-28

**Authors:** Rodrigo C Borges, Celso RF Carvalho, Alexandra S Colombo, Mariucha PS Borges, Francisco G Soriano

**Affiliations:** 1 University Hospital, University of São Paulo, SP, Brazil; 2 Physiotherapy Department, School of Medicine, University of São Paulo, SP, Brazil; 3 Internal Medicine Department, School of Medicine, University of São Paulo, SP, Brazil

## Introduction

Sepsis is a clinical problem of great relevance within the ICU because survivors can suffer from severe dysfunction and symptoms, such as fatigue, dyspnea, muscle weakness and a decrease in the health-related quality of life; however, the effects of this disease on physical activity in daily life in the short and medium term are not known.

## Objective

The objective of the study was to quantify the physical activity in daily life, muscle strength and exercise capacity in the short and medium term in survivors from severe sepsis and septic shock. Furthermore, we investigated clinical and laboratory factors that determine muscle strength, exercise capacity and physical activity in daily life.

## Methods

Prospective cohort study with a follow-up from hospital admission to 3 months after hospital discharge. Seventy-two patients admitted to the ICU due to severe sepsis or septic shock and a control group of healthy sedentary subjects (*n *= 50) were enrolled. All patients had their physical activity in daily life quantified by an accelerometer during their hospital stay and 3 months after hospital discharge. Exercise capacity (6-minute walking distance) and respiratory, handgrip and quadriceps muscle strength were also evaluated during hospitalization and 3 months after.

## Results

During hospitalization, patients spent the majority of their time inactive in a lying or sitting position (90 ± 34 % of daily time). Physical inactivity was partially reduced 3 months after hospital discharge (58 ± 20 % of daily time). However, the time patients spent walking was only 63 % of the time reported for healthy subjects. Patients also showed a reduction in walking intensity. At hospital discharge, muscle strength and exercise capacity were approximately 54 % of the predicted value, and these parameters showed a small but significant increase in patients 3 months after hospital discharge (70 % of predicted value). A multivariate regression analysis demonstrated that the use of systemic corticosteroids and hospitalization time negatively influenced quadriceps strength and exercise capacity at the time of hospital discharge.

## Conclusion

Our results strongly suggest that survivors of sepsis admitted to the ICU have a substantial reduction in physical activity, exercise capacity and muscle strength compared with healthy subjects that remains even 3 months after hospital discharge.

